# Marine compound rhizochalinin shows high *in vitro* and *in vivo* efficacy in castration resistant prostate cancer

**DOI:** 10.18632/oncotarget.11941

**Published:** 2016-09-10

**Authors:** Sergey A. Dyshlovoy, Katharina Otte, Winfried H. Alsdorf, Jessica Hauschild, Tobias Lange, Simone Venz, Christiane K. Bauer, Robert Bähring, Kerstin Amann, Ramin Mandanchi, Udo Schumacher, Jennifer Schröder-Schwarz, Tatyana N. Makarieva, Alla G. Guzii, Kseniya M. Tabakmakher, Sergey N. Fedorov, Larisa K. Shubina, Igor E. Kasheverov, Heimo Ehmke, Thomas Steuber, Valentin A. Stonik, Carsten Bokemeyer, Friedemann Honecker, Gunhild von Amsberg

**Affiliations:** ^1^ University Medical Center Hamburg-Eppendorf, Department of Oncology, Haematology and Bone Marrow Transplantation, Section Pneumology, Hubertus Wald -Tumorzentrum, Hamburg, Germany; ^2^ G.B. Elyakov Pacific Institute of Bioorganic Chemistry, Vladivostok, Russian Federation; ^3^ School of Natural Sciences, Far Eastern Federal University, Vladivostok, Russian Federation; ^4^ Department of Anatomy and Experimental Morphology, University Medical Center Hamburg-Eppendorf, Hamburg, Germany; ^5^ Department of Medical Biochemistry and Molecular Biology, University of Greifswald, Greifswald, Germany; ^6^ University Medical Center Hamburg-Eppendorf, Department of Cellular and Integrative Physiology, Hamburg, Germany; ^7^ Nephropathology Department, University Medical Center Erlangen, Erlangen, Germany; ^8^ Shemyakin-Ovchinnikov Institute of Bioorganic Chemistry, Moscow, Russia; ^9^ University Medical Center Hamburg-Eppendorf Martiniklinik, Prostate Cancer Center, Hamburg, Germany; ^10^ Tumor and Breast Center ZeTuP St. Gallen, St. Gallen, Switzerland

**Keywords:** rhizochalinin, castration resistant prostate cancer, AR-V7, apoptosis, autophagy

## Abstract

Development of drug resistance is an inevitable phenomenon in castration-resistant prostate cancer (CRPC) cells requiring novel therapeutic approaches. In this study, efficacy and toxicity of Rhizochalinin (Rhiz) – a novel sphingolipid-like marine compound – was evaluated in prostate cancer models, resistant to currently approved standard therapies. *In vitro* activity and mechanism of action of Rhiz were examined in the human prostate cancer cell lines PC-3, DU145, LNCaP, 22Rv1, and VCaP. Rhiz significantly reduced cell viability at low micromolar concentrations showing most pronounced effects in enzalutamide and abiraterone resistant AR-V7 positive cells. Caspase-dependent apoptosis, inhibition of pro-survival autophagy, downregulation of AR-V7, PSA and IGF-1 expression as well as inhibition of voltage-gated potassium channels were identified as mechanisms of action. Remarkably, Rhiz re-sensitized AR-V7 positive cells to enzalutamide and increased efficacy of taxanes.

*In vivo* activity and toxicity were evaluated in PC-3 and 22Rv1 NOD SCID mouse xenograft models using i.p. administration. Rhiz significantly reduced growth of PC-3 and 22Rv1 tumor xenografts by 27.0% (*p* = 0.0156) and 46.8% (*p* = 0.047) compared with controls with an increased fraction of tumor cells showing apoptosis secondary to Rhiz exposure. In line with the *in vitro* data, Rhiz was most active in AR-V7 positive xenografts *in vivo*. In animals, no severe side effects were observed.

In conclusion, Rhiz is a promising novel marine-derived compound characterized by a unique combination of anticancer properties. Its further clinical development is of high impact for patients suffering from drug resistant prostate cancer especially those harboring AR-V7 mediated resistance to enzalutamide and abiraterone.

## INTRODUCTION

Significant progress has been made in the treatment of castration-resistant prostate cancer (CRPC) in recent years. Docetaxel, cabazitaxel, abiraterone acetate, enzalutamide, sipuleucel T, and radium-223 all significantly improved overall survival in phase III clinical trials [3]. However, with time, most patients lose drug sensitivity revealed by a reduced progression-free survival with each additional treatment line [4]. Primary and secondary drug-resistance are responsible for this limitation of success [5]. Therefore, novel treatment options to overcome drug-resistance in CRPC are urgently needed.

**Figure 1:**
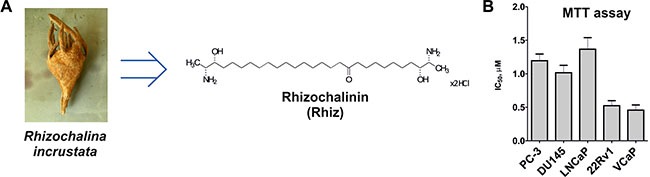
Effect of Rhiz on viability and proliferation of prostate cancer cells (**A**) The structure of Rhizochalinin (Rhiz, hydrochloride salt), derived from marine sponge *Rhizochalina incrustata*. (**B**) MTT assay: IC_50_ values (μM) of human prostate cancer cell lines treated with Rhiz for 48 h.

In the last decades, marine organisms have served as a source of new potent anticancer drugs. Interestingly, sessile marine sponges were found to produce a diversity of highly bioactive metabolites to protect themselves against predators. Eribulin, trabectedin, and monomethylauristatin E (MMAE) are recently approved marine-derived anticancer drugs. Their unique mechanisms of action explains their ability to overcome resistance against other chemotherapeutic agents [6, 7] Rhizochalinin (Rhiz, also referred to as aglycon of rhizochalin; Figure 1A) is a sphingolipid-like semi-synthetic compound. It is hydrolytically derived from rhizochalin [8] – a bioactive substance initially isolated from the marine sponge *Rhizochalina incrustata* (Figure 1A) [9]. Preliminary experiments showed cytotoxic effects of Rhiz on human cancer cell lines THP-1, HeLa, and SNU-C4 *in vitro* [10]. However, no detailed characterization of the marine compound has been carried out to date and *in vivo* data are pending.

In this study, we examined the mode of action of Rhiz in human prostate cancer models and evaluated its potential to overcome drug resistance. Additionally, first efficacy and toxicity data of the marine compound were generated *in vivo*.

## RESULTS

### Rhiz reduces prostate cancer cell viability and proliferation *in vitro*

Rhiz (Figure 1A) revealed cytotoxic effects on all human prostate cancer cell lines (PC-3, DU145, LNCaP, 22Rv1, VCaP) at low micromolar concentrations. Remarkably, the strongest cytotoxic effects were observed in AR-V7 positive (and therefore enzalutamide and abiraterone resistant [11]) 22Rv1 and VCaP cells. Dose-dependent cytotoxic effects of Rhiz were investigated by MTT assay. PC-3, DU145, and LNCaP cells showed comparable sensitivity towards Rhiz, whereas 22Rv1 and VCaP cells were significantly more sensitive compared to PC-3, DU145 and LNCaP (Figure 1B).

### Rhiz induces caspase-dependent apoptosis in prostate cancer cells

**Figure 2:**
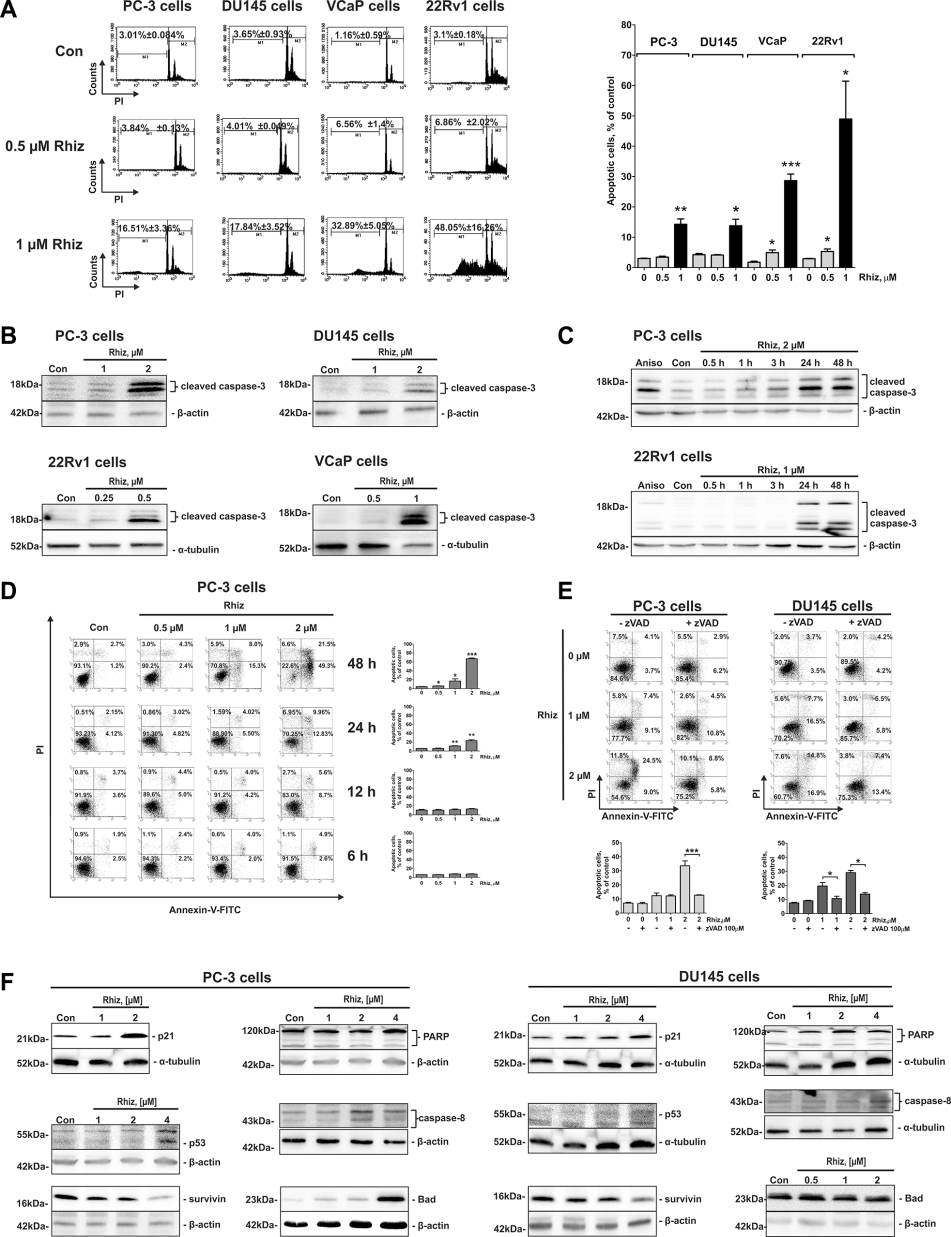
Effect of Rhiz on induction of apoptosis Effect of Rhiz (0.5 and 1 μM) on induction of apoptosis (**A**) after 48 h of treatment in PC-3, DU145, 22Rv1, and VCaP cells, analyzed by FACS. Apoptotic cells were detected as sub-G1 population in the cell cycle distribution histogram; black columns indicate percentage of apoptotic cells at 1 μM of Rhiz (A). (**B**) Western blotting (cropped blots), showing dose-dependent cleavage of caspase-3 in prostate cancer cells at the indicated cell line dependent threshold concentrations of Rhiz to start caspase-3 cleavage after 48 h of treatment. (**C**) Western blotting (cropped blots) showing time-dependent cleavage of caspase-3 in prostate cancer cells treated with the indicated concentrations of Rhiz for 0.5, 1, 3, 24, and 48 h. Cells treated with 5 μM of anisomycin for 48 h (Aniso) were used as a positive control. (**D**), (**E**), Flow cytometry analyses of induction of apoptosis in PC-3 and DU145 cells treated with Rhiz using double staining annexin-V-FITC/PI. (**D**) PC-3 cells were treated with Rhiz at indicated concentrations for 6, 12, 24 or 48 h, and total apoptotic (annexin-V-FITC-positive) cells were quantified. (**E**) PC-3 and DU145 cells were pre-treated with 100 μM of pan-caspase inhibitor zVAD for 1 h following treatment with indicated concentrations of Rhiz for 48 h. Total apoptotic cells were quantified. (**F**) Western blotting analysis (cropped blots) of expression of pro- and anti-apoptotic protein expression in prostate cancer cells treated with Rhiz for 48 h.

Hallmarks of apoptosis including increased sub-G1 cell population (Figure 2A), time- and dose-dependent caspase-3 cleavage (Figure 2B–2C), as well as phosphatidylserine externalization (Figure 2D–2E) were detected in Rhiz-treated cells. Pre-treatment with the pan-caspase inhibitor zVAD significantly decreased the apoptotic cell rate, indicating that the induction of apoptosis is caspase-dependent (Figure 2E). Remarkably, apoptosis induction was more pronounced in 22Rv1 and VCaP cells than in PC-3 and DU145 cells which is in line with the observed IC_50_ values revealing strongest cytotoxic effects of Rhiz in AR-V7 positive cell lines (Figure 2A).

Next, the impact of Rhiz on the expression of pivotal proteins involved in the regulation of apoptosis in prostate cancer cells was evaluated. Several pro-apoptotic proteins, such as p21, p53, Bad, cleaved-PARP and -caspase-8 were upregulated, while anti-apoptotic survivin was downregulated by Rhiz. No alterations of Bax, Pak1, Caspase-9 and Bcl-2 were found (data not shown). It should be noted that both PC-3 and DU145 cells bear mutated p53 [12]. Thus, a conclusion on the impact of p53 on the cytotoxic effect of Rhiz cannot be drawn.

### Rhiz inhibits autophagy in prostate cancer cells

**Figure 3:**
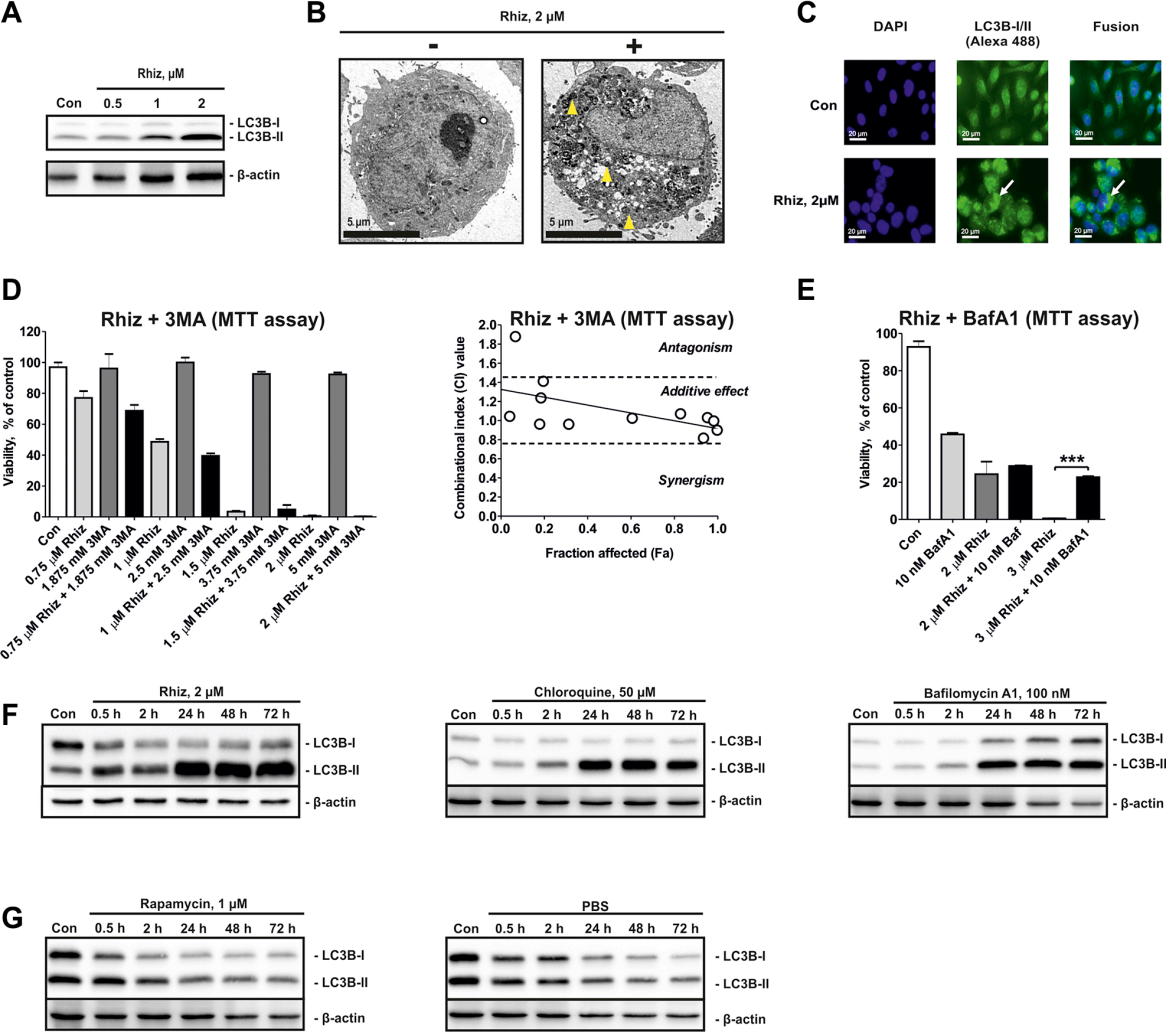
Hallmarks of autophagy inhibition in PC-3 cells treated with Rhiz (**A**) Effect of Rhiz on LC3B-I/II in PC-3 cells after 48 h of treatment. (**B**) Detection of autophagosome formation by electron microscopy. PC-3 cells were treated with Rhiz for 48 h. The number and size of autophagosomes (double membrane bound vesicles, indicated by yellow arrows) was significantly increased in treated cells. (**C**) Detection of autophagosomes by immunofluorescence microscopy. Cells were treated with 2 μM of Rhiz for 48 h, fixed, permeabilized, and treated with anti-LC3B-I/II antibody followed by treatment with Alexa Fluor 488-conjugated secondary antibody. The number of LC3B-I/II-positive organelles (autophagosomes, appeared as puncta and are indicated by arrows) is significantly increased in treated cells. (**D**), (**E**) Effect of Rhiz in combination with 3MA and BafA1 in PC-3 cells. Cells were co-treated with different concentrations of the single substances or their combination for 48 h. A constant molar ratio of the drugs ((**D**) ratio C(Rhiz):C(3MA) = 2:5000) or constant concentration of 10 nM BafA1 (**E**) was used. The combinational index (CI) values were calculated with CompuSyn Software. Cell viability was measured by MTT assay. (**F**), (**G**) Effect of Rhiz and other autophagy inhibitors and inducers on the LC3B-I/II level dynamics in PC-3 cells. Cells were exposed for 0.5 − 72 h to Rhiz, inhibitors of autophagy ((**F**) CQ, BafA1), or inducers of autophagy ((**G**) Rapa, starvation conditions – culture media was substituted with PBS). LC3B-I/II levels were detected by Western blotting (cropped blots).

The effects of Rhiz on autophagy-related processes were evaluated in PC-3 cells as described previously [13]. Protein expression analysis revealed a clear shift of the LC3B-I / LC3B-II ratio towards LC3B-II accumulation after 48 h of treatment (Figure 3A). In addition, formation and accumulation of autophagosomes were observed by electron microscopy (Figure 3B), as well as immunofluorescence analysis (intracellular LC3BI/II-positive structures, Figure 3C). In order to differentiate between autophagy inhibition and induction we investigated the effect of the autophagy inhibitor 3-methyladenine on Rhiz mediated cytotoxicity. Combining Rhiz and 3-methyladenine clearly showed additive effects in MTT-based Chou-Talalay assays (Figure 3D), suggesting that both compounds inhibit autophagy. In line with this finding, bafilomycin A1, known to antagonize cytotoxicity of other autophagy inhibitors [14] was able to inhibit cytotoxic effects of Rhiz (Figure 3E).

To examine a potential inhibitory effect of Rhiz on pro-survival autophagy flux we explored the kinetics of autophagosome formation-degradation using LC3B-I/II as a marker of this process. For cells treated with Rhiz and known late stage autophagy inhibitors like bafilomycin A1 and chloroquine, the LC3B-II level constantly increased (Figure 3F), whereas induction of autophagy by rapamycin or starvation resulted in constant downregulation of LC3B-II (Figure 3G). Thus, these results strongly suggest that Rhiz inhibits late stages of pro-survival autophagy in human CRPC cells.

### Rhiz blocks voltage-gated potassium channels

**Figure 4:**
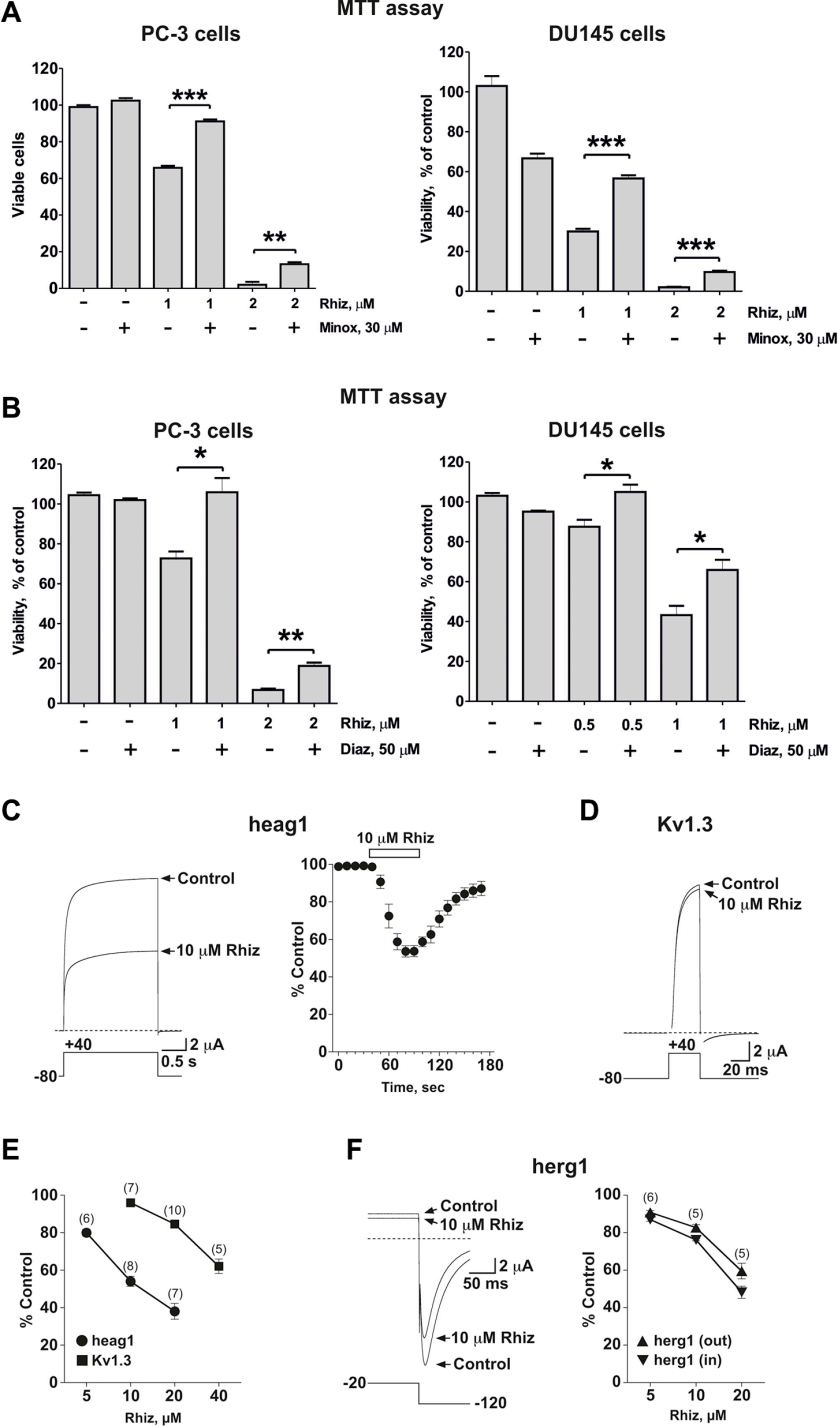
Inhibitory effect of Rhiz on potassium channels (**A**), (**B**) Effect of Rhiz in combination with potassium channel openers Minox (**A**) and Diaz (**B**) on viability of PC-3 and DU145 cells. Cells were co-treated with indicated concentrations of the drugs for 48 h. Cell viability was measured by MTT assay. (**C**–**F**) Effect of Rhiz on voltage-dependent potassium channels expressed in *Xenopus* oocytes. Currents mediated by heag1 (**C**) and Kv1.3 (**D**) and herg1 (**F**) are shown before and at the end of 10 μM Rhiz application. Dotted lines represent zero current. Pulse protocols are shown below traces. Time course and reversibility of current inhibition is shown for heag1 (**C**). (**E**), (**F**) Concentration-dependence of remaining current (% of control) in the presence of Rhiz for heag1 and Kv1.3 (**E**) and for herg1 ((**F**) outward and inward current) channels.

Potassium channels are involved in the metastatic spread of prostate cancer cells [15]. Thus, we examined the effect of minoxidil and diazoxide, two well-known potassium channel openers, on the cytotoxic effect of Rhiz (Figure 4A, 4B). Both drugs significantly inhibited the Rhiz-mediated reduction of cell viability. Consequently, we evaluated the effect of Rhiz on heag1 and Kv1.3, two voltage-gated potassium channels found in prostate cancer cells [15, 16]. In the *Xenopus* oocyte expression system, 10 μM Rhiz induced a half maximal inhibition of heag1-mediated currents in a fast and reversible manner (Figure 4C). Much higher concentrations of Rhiz were needed to effectively reduce Kv1.3-mediated currents (Figure 4D, 4E), and the inhibition of herg1 channels (also found in prostate cancer cells [17], but an undesired target in cardiopharmacology [18]) was weaker than the one observed for heag1 (Figure 4F). The inhibition of heag1 channels expressed in a mammalian cell line by 10 μM Rhiz was stronger (~80 % within 1 min; see Supplementary Figure S1) than in the *Xenopus oocyte* expression system.

### Rhiz significantly downregulates AR-V7, PSA and IGF-1 expression in prostate cancer cells

**Figure 5:**
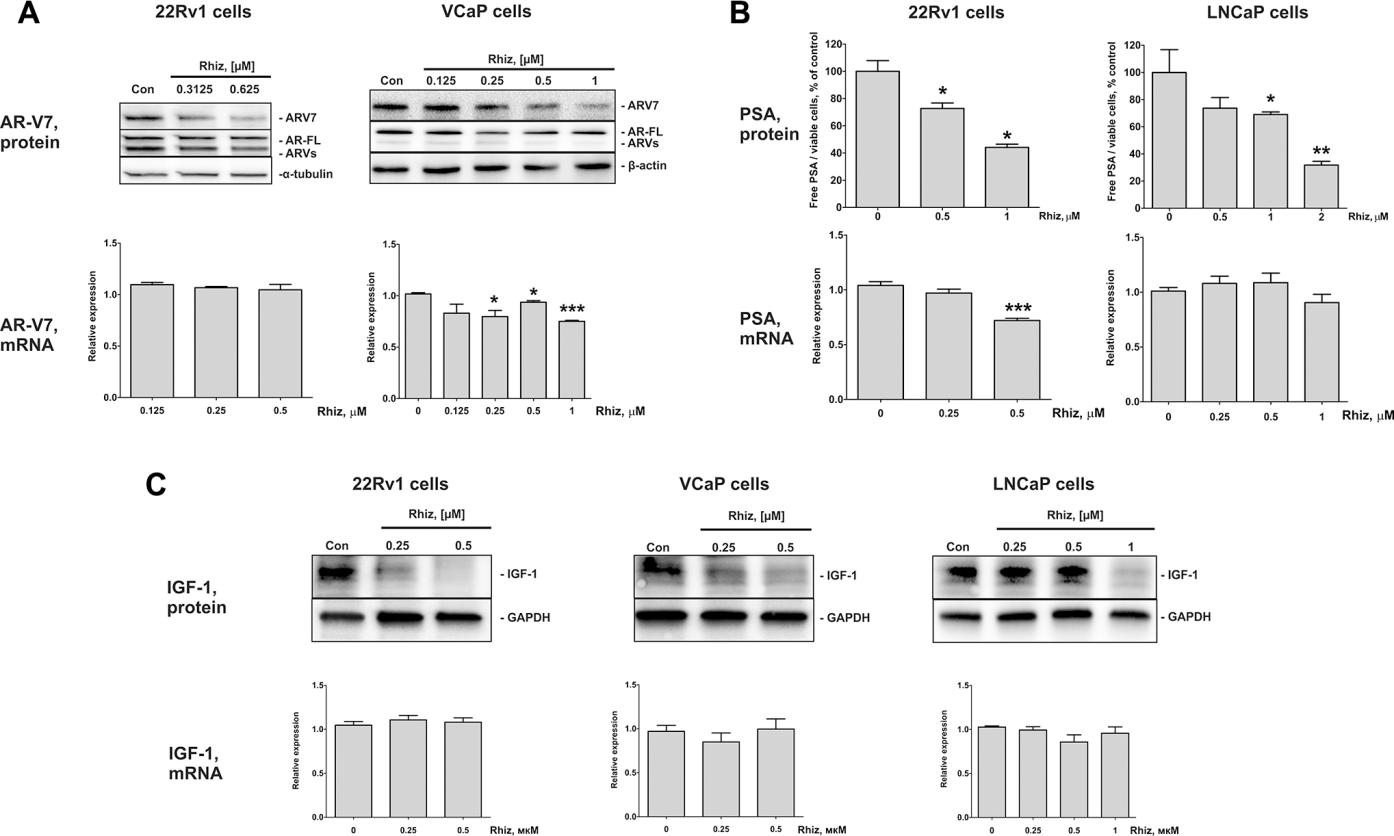
Effect of Rhiz on AR-V7, PSA, and IGF-1 protein and mRNA expression (**A**) Effect of Rhiz on AR-V7 expression in 22Rv1 and VCaP cells. Protein expression was analyzed by Western blotting (cropped blots), mRNA levels were analyzed by qPCR. (**B**) Effect of Rhiz on PSA expression in 22Rv1 and LNCaP cells. The concentration of PSA in the culture supernatant was analyzed using ELISA and normalized to the number of viable cells, mRNA levels were analyzed by qPCR. (**C**) Effect of Rhiz on IGF-1 expression in 22Rv1, VCaP, and LNCaP cells analyzed by Western blotting (cropped blots), mRNA levels were analyzed by qPCR.

The androgen receptor splice variant 7 (AR-V7) is associated with resistance to enzalutamide and abiraterone acetate, since it lacks the C-terminal ligand binding domain. Moreover, AR-V7 is a constitutively active transcription factor thus promoting prostate cancer cell growth and proliferation [19]. As described previously, the strongest cytotoxic effect of Rhiz was observed in the AR-V7 positive cell lines 22Rv1 and VCaP. Therefore, we investigated Rhiz ability to affect AR-V7 expression and AR-signaling on both protein and mRNA levels in 22Rv1, VCaP and LNCaP cells. Rhiz significantly downregulated AR-V7 in VCaP and 22Rv1 cells (Figure 5A), while the full-length androgen receptor (AR-FL) was not affected in either cell line. Additionally, Rhiz significantly decreased expression of PSA and IGF-1, two downstream targets of the AR, in 22Rv1 and LNCaP cells (Figure 5B, 5C). In VCaP cells Rhiz induced a significant downregulation of IGF-1 (Figure 5C), however no decrease of PSA was detected (data not shown).

The effect of Rhiz on the corresponding genes expression (mRNA levels) was examined using qPCR. Slight downregulation of AR-V7 mRNA was only observed in VCaP cells (Figure 5A), whereas PSA mRNA was found to be downregulated in 22Rv1 cells only (Figure 5B). No regulation of IGF-1 mRNA was observed in either 22Rv1, VCaP, or LNCaP cells (Figure 5C). Therefore, the molecular mechanisms of the effect on AR-signaling seem to be cell type-specific.

### Rhiz enhances cytotoxic effects of docetaxel and cabazitaxel and re-sensitizes AR-V7 positive cells to enzalutamide

**Figure 6:**
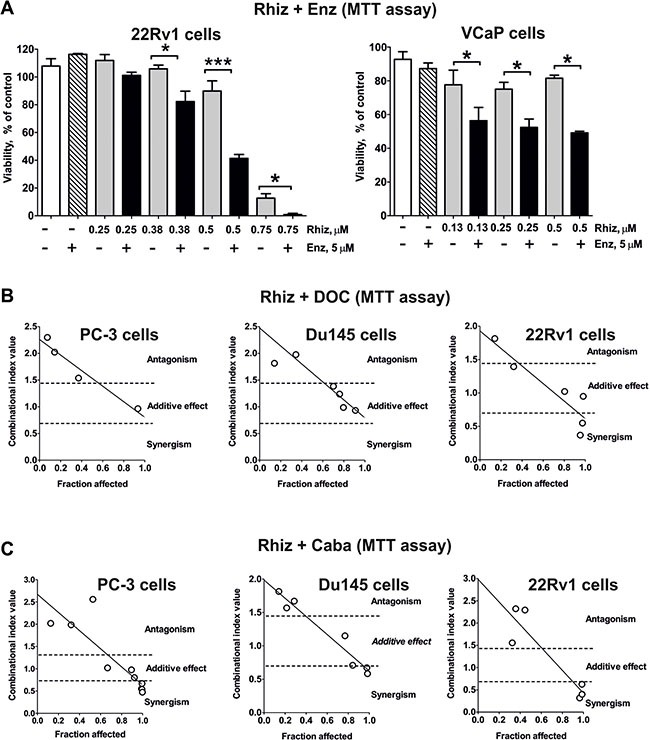
Effect of Rhiz on the cell viability in combination with other drugs (**A**) Effect of Rhiz in combination with Enz in 22Rv1 and VCaP cells. Cell viability was measured by MTT assay. In all experiments cells were treated for 48 h. (**B**), (**C**) Effect of Rhiz in combination with docetaxel (**B**) or cabazitaxel (**C**) on PC-3, DU145 or 22Rv1 cells, examined by MTT assay. Cells were co-treated with different concentrations of the single substances or their combination for 48 h at a constant molar ratio. The combinational index (CI) values were calculated with CompuSyn software. The molar ratio of the substances used for the combination C(Rhiz):C(DOC) was 100:1 (for PC-3 and DU145 cells) or 25:1 (for 22Rv1 cells); for combination C(Rhiz):C(Caba) the molar ratio was 25:1 (for PC-3 cells) or 125:2 (for DU145 and 22Rv1 cells). The raw CI values from several independent experiments for each combination are represented on the correspondent graphs.

Next, the effect of Rhiz in combination with standard therapies was examined. Interestingly, Rhiz was able to re-sensitize AR-V7-positive 22Rv1 and VCaP cells to enzalutamide (Figure 6A). In addition, Rhiz showed additive effects in combination with docetaxel or cabazitaxel at high values of Fa (fraction affected) (Figure 6B–6C).

### Dose-finding *in vivo* experiments

Extrapolating Rhiz IC_50_~1.5 μmol/L to the mouse model (=1.5 μmol/kg), a theoretical effective Rhiz concentration of 0.8 mg/kg/day was calculated. Dose finding studies were performed starting with this concentration followed by stepwise dosage increase if animals were not compromised. Up to a concentration of 2.2 mg/kg/day, stable body weights of the mice were observed, while a daily treatment with 2.4 mg/kg/day caused a significant and fast decrease of body weight. However, at doses of 2.2 mg/kg/day and 2.0 mg/kg/day, slight signs of distress (slowed movement) were observed directly after i.p. injections, while no other side effects were found. Consequently, a dose of 1.8 mg/kg/day was chosen for further studies.

### Rhiz suppresses primary tumor growth *in vivo* by inducing tumor-cell apoptosis

**Figure 7:**
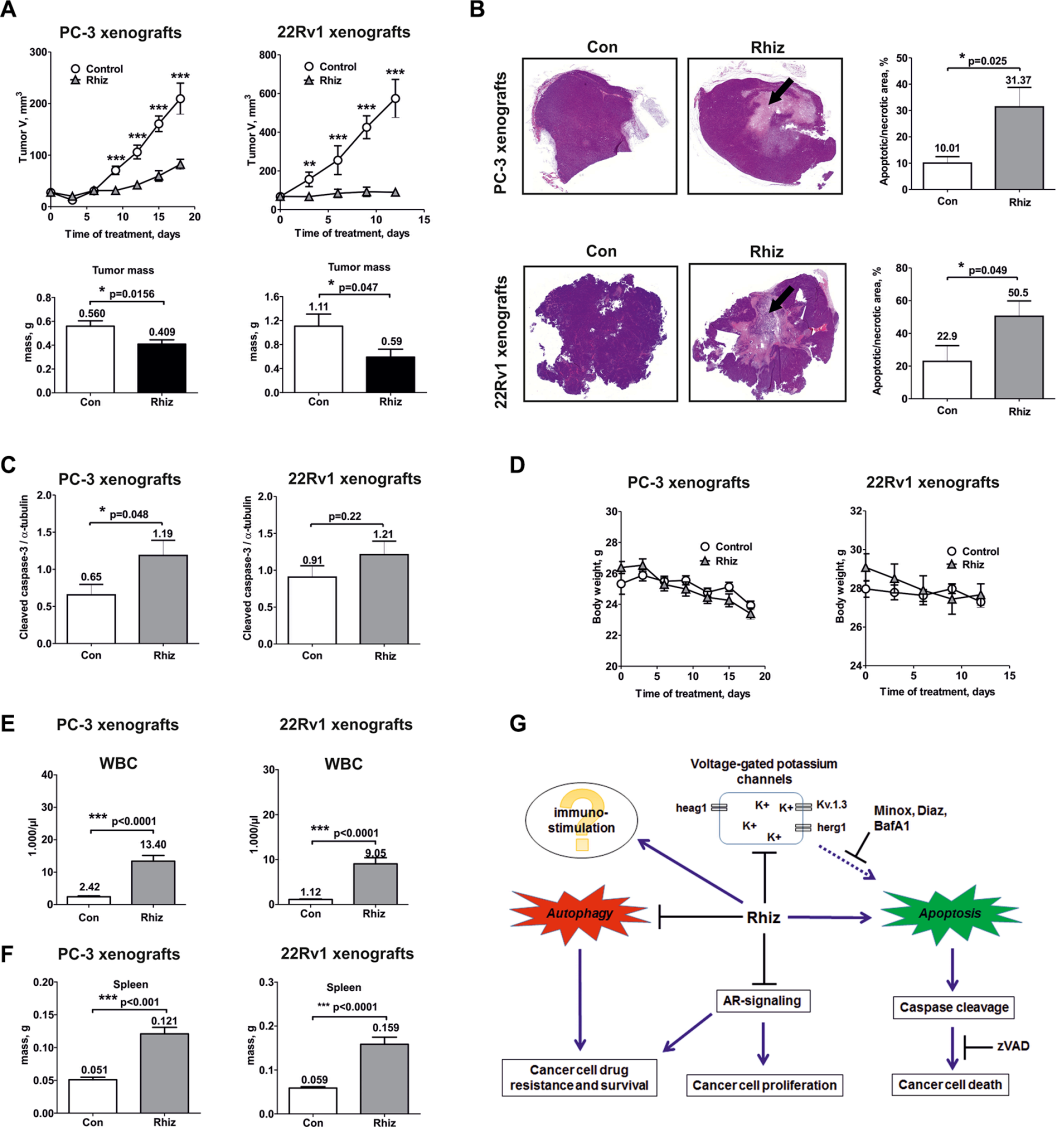
*In vivo* effect of Rhiz in a human prostate cancer xenograft model PC-3 and 22Rv1 cells were injected subcutaneously into male NOD SCID mice. Once primary tumors reached 50–60 mm^3^, daily treatment (1.8 mg/kg/day) was started. Control group was treated with placebo (0.9% NaCl). Solutions were administrated i.p. (**A**) Effect of Rhiz on tumor growth. The lower bar graphs represent tumor masses measured at the day of termination of the experiments after the animals were sacrificed. (**B**) Detection of tumor cell death *in vivo*. Histological sections of representative tumors from control and treated animals were stained with H&E. Quantification of dead cells was performed using ImageJ Software. Tumor necrotic/apoptotic areas are indicated by arrows. (**C**) Induction of caspase-3 cleavage in PC-3 and 22Rv1 cell xenografts was analyzed by Western blotting using protein extracts of tumor samples. (**D**) Effect of Rhiz on body weight. (**E**) Effect of Rhiz treatment on white blood cell (WBC) count in PC-3 and 22Rv1 cell xenografts. (**F**) Effect of Rhiz treatment on spleen weight in PC-3 and 22Rv1 cell xenografts. (**G**), Model of the supposed mode of anticancer action of Rhiz: induction of caspase-dependent apoptosis possibly through the inhibition of potassium channels, inhibition of pro-survival autophagy and AR-signaling, and possible immuno-stimulatory effect.

Efficacy and toxicity of Rhiz were investigated in PC-3 and 22Rv1 human xenograft tumor models. The experiments were performed with the well tolerated dose of 1.8 mg/kg/day. Rhiz significantly inhibited tumor growth and reduced tumor mass in both models upon daily i.p. administration, however, the strongest tumorsuppressive effect was observed in the AR-V7 positive cells xenograft model (Figure 7A).

The effect of Rhiz on the induction of apoptosis in human cancer cells *in vitro* was reassessed *in vivo* (Figure 7B, 7C). A significant increase of dead tumor cells was detected in PC-3 and 22Rv1 xenografts secondary to treatment with Rhiz by histological quantification of necrotic/apoptotic cells (Figure 7B). In addition, cleaved caspase-3 was significantly upregulated in PC-3 xenografts indicating that Rhiz induces caspase-dependent apoptosis of cancer cells *in vivo* (Figure 7C).

### Analysis of side effects

Overall, Rhiz was well tolerated at a dose of 1.8 mg/kg/day. No changes in mouse behavior, body weight, pain or distress were observed in any of the treated animals (Figure 7D). In blood analysis, thrombocytes and hemoglobin were within normal ranges (data not shown), while a significant increase of white blood cells (Figure 7E) with an absolute elevation of all leucocyte subclasses examined (monocytes, neutrophils, and lymphocytes, data not shown) was observed secondary to Rhiz treatment, suggesting a possible immune-stimulatory effect of Rhiz. In line with this, significant increase of the mean spleen weight was observed in PC-3 and 22Rv1 xenografts (Figure 7F). In addition, in one experiment a mild decrease of mean kidney weight was observed, while other organs were not significantly affected by Rhiz (data not shown).

## DISCUSSION

Development of resistance limits the success of currently approved drugs in CRPC [20]. To date, different mechanisms of action contributing to drug resistance have been identified, including increased drug efflux by p-glycoprotein [21], augmented cellular metabolism of drug detoxifying proteins, alterations in tubulin isotypes with different kinetics of microtubule formation [22], disturbance of AR trafficking [23] and induction of pro-survival autophagy [19, 24, 25]. In addition, AR-V7 was recently identified to cause resistance to AR-targeting drugs like abiraterone and enzalutamide due to a lack of the ligand binding domain [11].

Rhiz is a novel marine compound which significantly inhibits growth of different drug resistant CRPC cell lines *in vitro* and *in vivo* by a unique activity profile. It is capable to overcome two main mechanisms of drug-resistance in CRPC – pro-survival autophagy and AR-V7 expression. In order to reflect the diversity of castration resistant prostate cancer, the anticancer effect of Rhiz was explored using a panel AR/AR-V7 positive and AR negative CRPC cell lines.

According to recent studies several anticancer drugs including enzalutamide can induce pro-survival autophagy, resulting in increased cancer cell survival and thus mediating drug resistance [26]. In contrast, autophagy inhibitors demonstrated *in vitro* and *in vivo* efficacy in prostate cancer models [26]. In our study, Rhiz clearly inhibited late stages of pro-survival autophagy in human CRPC cells.

In addition, Rhiz was able to overcome AR-V7 mediated drug-resistance. In fact, Rhiz revealed strongest *in vitro* and *in vivo* activity in prostate cancer cells expressing high levels of AR-V7. Due to this we investigated Rhiz effect on AR-V7, PSA, and IGF-1 expression. Remarkably, Rhiz was found to downregulate AR-V7 in 22Rv1 and VCaP cells. Furthermore, expression of PSA and IGF-1, two main downstream targets of the AR, were decreased by Rhiz in 22Rv1 and LNCaP cells suggesting that Rhiz exerts its action by interacting with the androgen receptor pathway. In VCaP cells, only IGF-1 was downregulated, while PSA expression was not significantly influenced by Rhiz.

As cytotoxic effects of Rhiz were most pronounced in AR-V7-positive cells, we assume that apart from inducing apoptosis, inhibiting pro-survival autophagy and blocking potassium channels, the downregulation of AR-V7 and AR-target genes substantially contributes to Rhiz ability to inhibit prostate cancer cell growth and proliferation. Interestingly, Rhiz re-sensitized AR-V7-positive CRPC cells towards enzalutamide which can be most likely explained by the detected AR-V7 downregulation and unaffected AR-FL expression thus enabling enzalutamide to exert its action by binding to AR-FL. These data suggest that Rhiz may specifically or non-specifically target AR-V7. We therefore postulate that Rhiz main potential lies in providing a treatment option for CRPC patients with enzalutamide or abiraterone resistance due to its ability to downregulate AR-V7 expression thus mediating resensitivation towards both drugs. However, it should be noted that the molecular mechanisms of AR-signaling alteration by Rhiz seem to be cell type-dependent.

Moreover, Rhiz enhanced cytotoxic effects of cabazitaxel and docetaxel. However, this effects may be non-specific for the taxanes. Thus, combinations with other cytotoxic agents are currently investigated. Furthermore, Rhiz effectively blocked potassium channels. Notably, current inhibition of the potassium channels heag1, herg1, and Kv1.3 was observed immediately after application of Rhiz, suggesting a direct molecular effect of the drug on these channels. In prostate cancer, overexpression of potassium channels has been associated with high proliferation rates of tumor cells [15, 27]. In fact, in our experiments, induction of apoptosis by Rhiz was inhibited by potassium channel openers. Additionally, bafilomycin A1 reduced the cytotoxic effects of Rhiz presumably due to its ability to antagonize the cytotoxic effects of autophagy inhibitors [14] and to serve as a potassium-ionophore mediating K^+^-efflux [28].

To promote clinical development of a drug, evaluation of *in vivo* efficacy and toxicity are essential steps. Thus, dose-finding experiments were carried out and a well tolerable dose was identified. Remarkably, Rhiz significantly inhibited tumor growth of CRPC *in vivo* without treatment-limiting side effects at the chosen dose. However, a significant increase of spleen size and leukocytosis was detected in both experiments, suggesting an immune-stimulatory effect of the drug. In accordance with the *in vitro* data Rhiz induced caspase-dependent apoptosis *in vivo.*

Taken together, Rhiz is a promising marine-derived compound showing high *in vitro* and *in vivo* activity in AR-V7-positive and -negative CRPC cell lines. The compound overcomes drug resistance, re-sensitizes CRPC cells to enzalutamide and enhances the effect of taxanes. The mode of action of Rhiz comprises caspase-dependent apoptosis, inhibition of pro-survival autophagy, suppression of AR-signaling, and potentially immune-stimulatory effects (Figure 7G). Additionally, voltage-gated potassium channels have been identified as a molecular target of Rhiz. This unique combination of anticancer properties makes Rhiz a promising drug for the treatment of CRPC. Given the convincing *in vivo* activity and safety profile of the compound, further clinical development, namely chemical synthesis of Rhiz from commercially available reagents as well as “hit to lead” (structure optimization) drug discovery step, is currently under development.

## MATERIALS AND METHODS

### Reagents and antibodies

Rhizochalinin (Rhiz, rhizochalin aglycon, Figure 1A), was synthesized from rhizochalin through hydrolysis as reported previously [8]. For *in vitro* experiments the 2 mM stock solution of Rhiz in DMSO was used. The purity of the compound was verified by HPLC, ^1^ H and ^13^ C NMR spectroscopy. Anisomycin and docetaxel (10 mg/mL) were purchased from NeoCorp (Weilheim, Germany), 3-methyladenine and z-VAD(OMe)-fmk from Enzo Life Sciences (Farmingdale, NY, USA). Matrigel was purchased from BD Biosciences (San Jose, CA, USA); MTT (3-(4,5-dimethylthiazol-2-yl)-2,5-diphenyltetrazolium bromide) reagent, propidium iodide (PI) and chloroquine from Sigma (Taufkirchen, Germany); annexin-V-FITC from BD Bioscience (San Jose, CA, USA); enzalutamide and minoxidil from Selleckchem (Munich, Germany); diazoxide from R&D Systems (Wiesbaden, Germany); bafilomycin A1 and rapamycin from LC Laboratories (Woburn, MA, USA); cabazitaxel (10 mg/mL) was provided by Sanofi (Paris, France). Antibodies used were obtained commercially and are listed in Supplementary information (Supplementary Table S1). The current research was performed according to the Good laboratory practice regulations (GLPs).

### Cell lines and culture conditions

The human prostate cancer cell lines PC-3, DU145, 22Rv1, VCaP, and LNCaP were obtained from ATCC (Manassas, VA, USA). In literature PC-3 cells are described to be docetaxel-resistant [29]. All cell lines except LNCaP cells are androgen-independent and abiraterone/enzalutamide-resistant due to the absence of AR (PC-3 and DU145) or the presence of AR-V7 (22Rv1 and VCaP). Cells were incubated at 37°C in a humidified atmosphere with 5% (v/v) CO_2_. Cells were continuously kept in culture for a maximum of 3 months, and were routinely inspected microscopically for stable phenotype and regularly checked for contamination with mycoplasma. All cell lines were recently authenticated by a commercial service (Multiplexion, Heidelberg, Germany) using single nucleotide polymorphism (SNP)-profiling method.

PC-3, DU145 and 22Rv1 cells were cultured in 10% FBS/RPMI medium (RPMI medium supplemented with Glutamax^TM^-I (Invitrogen, Paisley, UK) containing 10% fetal bovine serum (FBS, Invitrogen) and 1% penicillin/streptomycin (Invitrogen)). LNCaP cells were cultured in 10% FBS/RPMI medium (RPMI medium supplemented with Glutamax^TM^-I containing 10% FBS, 1% penicillin/streptomycin, and 1 mM sodium pyruvate (Invitrogen)). VCaP cells were cultured in 10% FBS/DMEM medium (DMEM medium supplemented with Glutamax^TM^-I (Invitrogen) containing 10% FBS and 1% penicillin/streptomycin (Invitrogen)).

### *In vitro* cell viability assays

Cytotoxicity profiles of single compounds and drug combinations were evaluated by MTT or trypan blue-based viability assays as described previously [30]. Duration of treatment was 48 h, unless otherwise stated.

### Examination of synergistic/antagonistic effects of drug combinations

Determination of synergistic, antagonistic, or additive effects of drugs used in combination assays was performed using the Chou-Talalay method as previously described [31]. Drugs (rizochalinin, autophagy inhibitors, potassium channel openers, androgen receptor targeting drugs, taxanes) were combined in a constant molar ratio as indicated in the figure legends. Data were generated by MTT assay. The combinational index (CI) was calculated with CompuSyn v.1.0. Software (ComboSyn, Inc., Paramus, NJ, USA). Fa (fraction affected) is defined as non-survival fraction at a certain dose of drugs or their combinations. Synergism is defined as a CI < 0.7, whereas antagonism has CI > 1.45, and a CI of 0.7~1.45 is considered an additive effect.

### Flow cytometry

Apoptosis induction was examined by flow cytometry using annexin-V-FITC/PI double staining, or PI staining of DNA, respectively. The experiments were performed as described previously [31].

### Protein expression analysis

Intracellular protein expression was analyzed using Western blotting. Cell treatment, protein extraction and Western blotting were performed as described previously [30]. Tumor samples were flash-frozen directly after tumor excisions and homogenized on ice prior to protein extraction. The antibodies used are listed in Supplementary information (Supplementary Table S1).

### Quantitative real-time PCR (qPCR)

Cells were seeded in Petri dishes (4 × 10^6^ cells per ø 10 cm dish in 10 mL of media for 22Rv1 and LNCaP cells; or 2 × 10^6^ cells per ø 6 cm dish in 5 mL of media for VCaP cells). After incubation overnight the media was replaced with the fresh corresponding media containing Rhiz at the different concentrations. After incubation for 48 h both alive and dead floating cells were harvested by scratching, pelleted, and homogenized using Tissue and Cell Homogenizer Kit (QIAshredder, Cat. # 79654, QIAGEN, Hilden, Germany), and the total RNA was isolated using PureLink^**®**^ RNA Mini Kit (Cat. # 12183018A, Invitrogen, Carlsbad, CA, USA) with the on-column DNA digestion using PureLink™ DNase (Cat. # 12185-010, Invitrogen). RNA was diluted up to 50 μL and its concentrations were measured. Then 1 μg of RNA for 22Rv1 or LNCaP cells, or 0.5 μg for VCaP cells were transcribed into cDNA using Maxima First Strand cDNA Synthesis Kit for RT-qPCR, with dsDNase (Cat. # K1671, Thermo Scientific, Vilnius, Lithuania) and the qPCR was performed using 2X KAPA SYBR FAST qPCR Master Mix Optimized for Roche LightCycler 480 (Cat. # KK4609, KAPA biosystems, Worburn, MA, USA) according to the manufacturer's instructions. 2 pmol of primers and 10 ng (for 22Rv1 or LNCaP cells) or 5 ng (for VCaP cells) of template cDNA were used per one reaction. Expression of human AR-V7, IGF-1, PSA, and GAPDH were analyzed using the specific primers (for primers sequence see Supplementary information, Supplementary Table S2), synthesized by Eurofins MWG-Biotech AG (Ebersberg, Germany). The PCR conditions were 30 sec 95°C, followed by 40 cycles of 15 sec 95°C, 5 sec 60°C, and 26 sec 72°C (measurement of fluorescence). Melting curve analysis (10 sec 95°C, 60 sec 65°C and 1 sec 97°C) was performed directly after PCR run. Relative expression was calculated using the 2^−ΔΔCT^ method. To test statistical significance, data were analyzed by unpaired Student's *t*-tests.

### Analysis of PSA expression

22Rv1 cells (0.4 × 10^6^ cells/well) were seeded in 6-well plates, incubated overnight, and the media was replaced with fresh media (2 mL/well) containing drugs in different concentrations and treated with Rhiz for 48 h. Then aliquots of 100 μL of the culture media (out of 2 mL media/well of 6-well plate) was centrifuged at 1500 rpm for 5 min and the extracellular expression of human prostate-specific antigen (PSA) was measured in the supernatant by ELISA using the ProStatus^TM^ PSA Free-/Total DELFIA^®^ Kit (PerkinElmer, Turku, Finland). PSA concentration was normalized to the number of viable cells in the correspondent wells, which was directly measured by trypan blue-based viability assays.

### Electrophysiological experiments

Human ether-à-go-go 1 (heag)1, human ether-à-go-go-related gene- (herg) 1 and human Kv1.3 potassium channels were expressed in *Xenopus* oocytes. Frogs were anesthetized in ethyl 3-aminobenzoate methanesulfonate (Sigma; 1.2 g / liter tap water), and part of the ovary lobes was surgically removed. The tissue was digested for 3–5 h in a calcium-free solution containing 82.5 mM NaCl, 2 mM KCl, 1 mM MgCl_2_, 5 mM HEPES, and 1.3 mg/mL collagenase type II (Biochrom), pH 7.5 with NaOH. Defolliculated stage V - VI oocytes were selected the next day, and 50 nl cRNA solution were injected per oocyte using a nanoliter 2000 microinjector (WPI). Amounts of cRNA were 5 ng per oocyte for heag1 and herg1, and 1 ng per oocyte for Kv1.3. Injected oocytes were incubated at 16°C in a solution containing 75 mM NaCl, 5 mM Na-pyruvate, 2 mM KCl, 2 mM CaCl_2_, 1 mM MgCl_2_, 5 mM HEPES, and 50 μg/mL gentamicin (Sigma), pH 7.5 with NaOH, and used for recordings after 1–3 days. Electrophysiological recordings were done with the two-electrode voltage-clamp technique using a TurboTec-03X amplifier (npi) and PatchMaster Software (HEKA). The holding potential was −80 mV. Heag1 and Kv1.3 channels were activated by voltage jumps from −80 mV to +40 mV, herg1 channels by voltage jumps from −80 mV to +20 mV (2 s), followed by a −20 mV interpulse (500 ms) and a −120 mV tail pulse. Test pulses were applied every 10 s for heag1 and herg1, and every 40 s for Kv1.3. Oocytes were bathed in a sodium-free (to minimize inward leak at holding potential) control solution containing 91 mM N-methyl-D-glucamine (NMDG), 5 mM KCl, 1 mM CaCl_2_, 1 mM MgCl_2_, 5 mM HEPES; pH 7.4 (HCl). The effect of Rhiz on potassium currents was tested by exchanging, during continuous test pulse application and recording, the control solution for one containing 5, 10, 20 or 40 μM of the substance for 60–90 s, before washing with control solution.

The effect of 10 μM Rhiz was also tested on heag1-mediated currents recorded from transiently transfected Chinese Hamster Ovary (CHO) cells (Supplementary Figure S1). CHO cells were grown with minimal essential medium (MEM) and transfected with heag1 cDNA (200 ng per 35 mm dish) using Lipofectamine reagent (Life Technologies) according to the manufacturer's instructions. CHO cells were used for electrophysiological recordings within 3 to 28 h after transfection. The bath solution contained 140 mM NaCl, 5 mM KCl, 1 mM CaCl_2_, 0.8 mM MgCl_2_, 10 mM HEPES, 5 mM Glucose; pH 7.4, (NaOH). Patch pipets, pulled from thin-walled borosilicate glass with a Zeitz universal puller and filled with internal solution containing 140 mM KCl 140, 1 mM CaCl_2_, 2 mM MgCl_2_, 2.5 mM EGTA, 10 mM HEPES; 7.35 pH (KOH), had bath resistances between 1.8 and 2.5 MΩ. Recordings were done in the whole-cell configuration of the patch-clamp technique using an EPC9 patch-clamp amplifier controlled with PULSE software. All electrophysiological recordings were measured at room temperature, and no leak subtraction was performed. Data were analyzed with PulseFit and FitMaster (HEKA).

### Microscopy

To confirm autophagosome accumulation, immunofluorescence and electron microscopy was applied as described previously [31]. To assess the formation of LC3-I/II-positive cellular structures by immunofluorescence analysis, PC-3 cells were pre-incubated overnight in 8-chamber glass slides (5 × 10^4^ cells/chamber). The medium was changed with medium containing Rhiz (2 μM). After 48 h of incubation cells were fixed and permeabilized as described previously [31]. After washing with PBS, samples were treated with 1:400 rabbit anti-LC3B-I/II antibody solution (in 0.1% (w/v) NaN_3_; 0.2% (w/v) BSA in PBS, pH 7.4) overnight at 4°C, washed with PBS and incubated with secondary anti-rabbit Alexa Fluor 488-conjugated antibody solution in PSB for 1 h at RT. Then, samples were washed with PBS, covered with DAPI-based ProLong^®^ Gold reagent (Life Technologies) and directly analyzed with AxioScope.A1 (Carl Zeiss) microscope at ×1000 magnification and with the AxioVision40 V4.8 software (Carl Zeiss Imaging Solutions). For electron microscopy, untreated and treated PC-3 cells were fixed using glutaraldehyde, and embedded in Epon-Araldite. Then, semi-thin and ultra-thin sections were cut and analyzed using a Zeiss microscope EM 906 (Carl Zeiss, Oberkochen, Germany) at various magnifications. To confirm cancer cell death *in vivo*, analyses of H&E stained tumor sections were performed as previously described [32].

### Subcutaneous xenograft mouse models

All animal experiments were approved by the local regulatory authorities (project No. G33/15). Dose-finding studies were carried out in healthy mice with a stepwise increase of Rhiz to determine the maximum tolerated dose. Mice were monitored daily for possible side effects. In a next step, PC-3 or 22Rv1 cells were s.c. xenotransplanted in male *Mus musculus* NOD SCID mice. Animals (male *Mus musculus* NOD SCID mice, age of 8–12 weeks, 20–25 g), were purchased from Charles River Laboratories. Animals were maintained in the animal facilities of the University Medical Center Hamburg-Eppendorf (Hamburg, Germany) under pathogen-free conditions in individually ventilated cages and fed with sterile standard food and water *ad libitum*.

For xenotransplantation, PC-3 and 22Rv1 cells were resuspended in FBS- and antibiotics-free RPMI media and mixed with matrigel (1:1 for PC-3 cells; 2:1 for 22Rv1 cells). Next, 200 μL of cell-matrigel suspension containing 1 × 10^6^ of PC-3 cells or 2 × 10^6^ of 22Rv1 cells were injected s.c. per mouse.

Once primary tumors reached 50–60 mm^3^ (7 days after PC-3 or 15 days after 22Rv1 cell xenotransplantation), animals were randomly assigned to a treatment and a control arm (10 mice per group), and therapy was initiated. Rhiz solution in 0.9% NaCl was daily administrated i.p. at the primary identified dose of 1.8 mg/kg/day Rhiz. The injection side (right or left lower part of the abdomen) was changed every day. Mice of the control groups received 0.9% NaCl. The volume of injections was calculated as 10 μL per 1 g of body weight for both treated and control groups. Common behavior (eating, drinking, climbing, social interactions) and signs of pain and distress (weight loss, ruffled fur, ocular discharge, lethargy, ataxia, labored respiration, hypothermia) were regularly recorded. Tumor volume and body weight were measured every 3–4 days. When primary tumors exceeded 2 cm^3^ or ulcerated, mice were terminally anesthetized, blood samples (~100 μL) were taken and the animals were sacrificed.

Tumors were divided into two pieces and fixed *en bloc* in formalin, or flash-frozen for further protein extraction as previously described [32]. The blood was analyzed with a HemaVet 950FS automatic veterinary hematology analyzer (Drew Scientific, INC, France) according to the manufacturer's protocol. Analysis of H&E stained tumor sections was performed by two independent blinded investigators for 5 tumors from each group (two biggest tumors + two smallest tumors + one middle size tumor from each group in both experiments). The quantification of necrotic-apoptotic area was performed using the Image J Software (NIH, Bethesda, USA) and is shown in Supplementary Figure S2 (Supplementary information). Activation (cleavage) of caspase-3 was measured in the tumor samples by Western blotting (Supplementary Figure S3). Tumor samples were homogenized with the blender, proteins were extracted with Western blotting lysis buffer, and 30 μg/slot of total protein extract was loaded. The level of cleaved caspase-3 was normalized against loading control. The signals within each membrane were normalized against the same control sample (which was loaded on each membrane) and therefore were compared within the different membranes.

### Statistical analyses

Statistical analyses were performed using GraphPad Prism software v.5.01 (GraphPad Prism Software Inc., La Jolla, CA, USA) and Stata 11 (StataCorp LP, TX, USA). Data are presented as mean ± SEM (standard error of the mean). Experiments were performed in triplicates and repeated at least three times unless stated otherwise. The unpaired Student's *t-test* was used for comparison between two groups. Differences were considered to be statistically significant if *p <* 0.05 (*p < 0.05, **p < 0.01, ***p < 0.001).

## SUPPLEMENTARY MATERIALS



## Abbreviations

Aniso: Anisomycin
AR: Androgen receptor
AR-V7: Androgen receptor splice variant 7
Ara-C: Arabinofuranosyl cytidine
22Rv1: ADT-resistant, AR/AR-V7 positive prostate cancer cell line [1]
3MA: 3-Methyladenine
ADT: Androgen deprivation therapy
ATCC: American type culture collection
BafA1: Bafilomycin A1
CQ: Chloroquine
Caba: Cabazitaxel
DOC: Docetaxel
DU145: ADT-resistant, AR-negative prostate cancer cell line [2]
Diaz: Diazoxide
Enz: Enzalutamide
Fa: Fraction affected (non-survival fraction at a certain drug dose)
H&E: Hematoxylin and eosin stain
IHC: Immuno-histochemical
LC3B: Microtubule-associated protein light chain 3
LNCaP: ADT-sensitive, AR-positive prostate cancer cell line [2]
Minox: Minoxidil
mCRPC: Metastatic castration resistant prostate cancer
MMAE: Monomethyl Auristatin E
PC-3: ADT-resistant, AR-negative prostate cancer cell line [2]
PSA: Prostate specific antigen
Rhiz: Rhizochalinin
VCaP: ADT-resistant, AR/AR-V7 positive prostate cancer cell line [1]
zVAD: z-VAD(OMe)-fmk.
