# Supplementary material for: Marine compound rhizochalinin shows high in vitro and in vivo efficacy in castration resistant prostate cancer
Source: Oncotarget. 2016 Sep 10;7(43):69703–17. doi: 10.18632/oncotarget.11941 (PMC5342509; doi:10.18632/oncotarget.11941)
Supplement: Supplementary file 1 [file oncotarget-07-69703-s001.pdf]

Marine compound rhizochalinin shows high *in vitro* and *in vivo* efficacy in castration resistant prostate cancer

Supplementary Materials

Supplementary Table S1: List of antibodies used

Antibodies	Clonality	Source	Cat.-No.	Used concentration	Manufacturer
anti-α-Tubulin	mAb	mouse	T5168	1:5000	Sigma-Aldrich
anti-β-Actin-HRP	pAb	goat	sc-1616	1:10000	Santa Cruz
anti-AR	pAb	rabbit	sc-816	1:200	Santa Cruz
anti-AR-V7	mAb	rabbit	198394	1:1000	abcam
anti-Bad	mAb	rabbit	#9239	1:1000	Cell Signaling
anti-Bax	mAb	rabbit	#5023	1:1000	Cell Signaling
anti-Bcl-2	pAb	rabbit	#2876	1:1000	Cell Signaling
anti-GAPDH	mAb	mouse	G041	1:10000	Abm
anti-Caspase-8	mAb	mouse	#9746	1:1000	Cell Signaling
anti-Caspase-9	pAb	rabbit	sc-8355	1:200	Santa Cruz
anti-cleaved Caspase-3	mAb	rabbit	#9664	1:1000	Cell Signaling
anti-IGF-1	mAb	mouse	sc-74116	1:500	Santa Cruz
anti-LC3B-I/II	pAb	rabbit	#2775	1:1000	Cell Signaling
anti-mTOR	mAb	rabbit	#2983	1:1000	Cell Signaling
anti-p21$^{Waf1/Cip1}$	mAb	rabbit	#2947	1:1000	Cell Signaling
anti-p53	mAb	mouse	#2524	1:1000	Cell Signaling
anti-PAK1	pAb	rabbit	#2602	1:1000	Cell Signaling
anti-PARP	pAb	rabbit	#9542	1:1000	Cell Signaling
anti-phospho-mTOR	mAb	rabbit	#5536	1:1000	Cell Signaling
anti-PTEN	mAb	mouse	04-035	1:1000	Millipore
anti-Survivin	pAb	rabbit	NB500-201	1:1000	Novus
anti-rabbit IgG-Alexa Fluor® 488		goat	#4412	1:1000	Cell Signaling
anti-goat IgG-HRP		rabbit	#31433	1:10000	Thermo Scientific
anti-mouse IgG-HRP		sheep	NXA931	1:10000	GE Healthcare
anti-rabbit IgG-HRP		goat	#7074	1:5000	Cell Signaling
anti-rat IgG-HRP		rabbit	ab6734-1	1:5000	abcam

Supplementary Table S2: List of primers used

Primer name	Primer sequence
AR-V7 forward	5′-CCA TCT TGT CGT CTT CGG AAA TGT TA-3′
AR-V7 reverse	5′-TTT GAA TGA GGC AAG TCA GCC TTT CT-3′
PSA forward	5′-CAT CAG GAA CAA AAG CGT GA-3′
PSA reverse	5′-ATA TCG TAG AGC GGG TGT GG-3′
IGF-1 forward	5′-AGG AAG TAC ATT TGA AGA ACG CAA GT-3′
IGF-1 reverse	5′-CCT GCG GTG GCA TGT CA-3′
GAPDH forward	5′-TGC ACC ACC AAC TGC TTA-3′
GAPDH reverse	5′-GAG GCA GGG ATG ATG TTC-3′

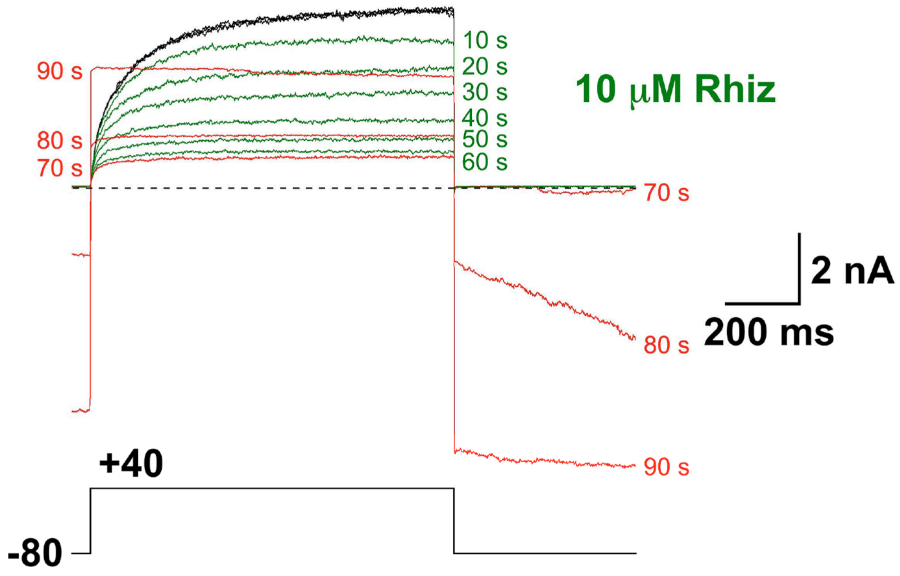

Supplementary Figure S1: Effect of Rhiz on heag1-mediated currents in a CHO cell. Currents in response to repeated depolarizing voltage jumps (pulse protocol below traces) were recorded with the whole-cell patch-clamp technique from a transiently transfected CHO cell expressing heag1 channels (3 superimposed black traces: control bath solution, colored traces: bath solution containing 10 μM Rhiz). Time after the start of Rhiz application is indicated. During the first 60 s onset of current inhibition is observed (green traces), which is then accompanied by considerable leak current development (zero current indicated by dotted line) and eventually loss of membrane integrity (red traces).

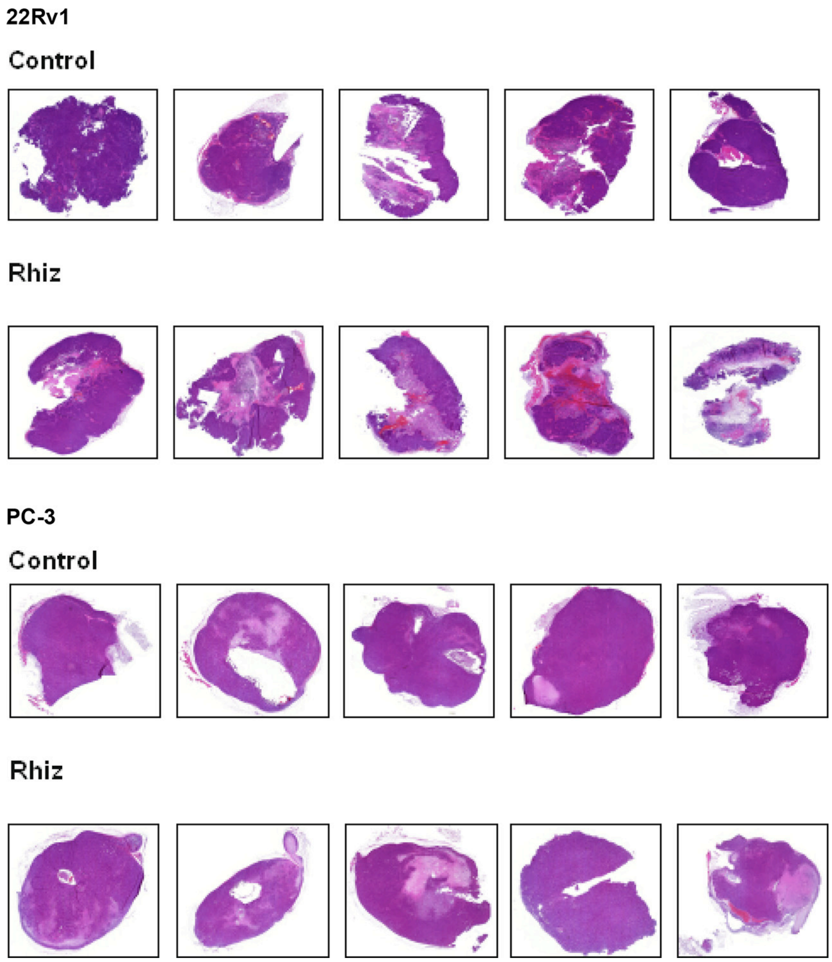

Supplementary Figure S2: H&E staining of the tumor sections. Analysis of H&E stained tumor sections was performed by two independent blinded investigators for 5 tumors from each group (two biggest tumors + two smallest tumors + one middle-sized tumor from each group in both experiments). The quantification of necrotic-apoptotic area was performed using the Image J Software (NIH, Bethesda, USA).

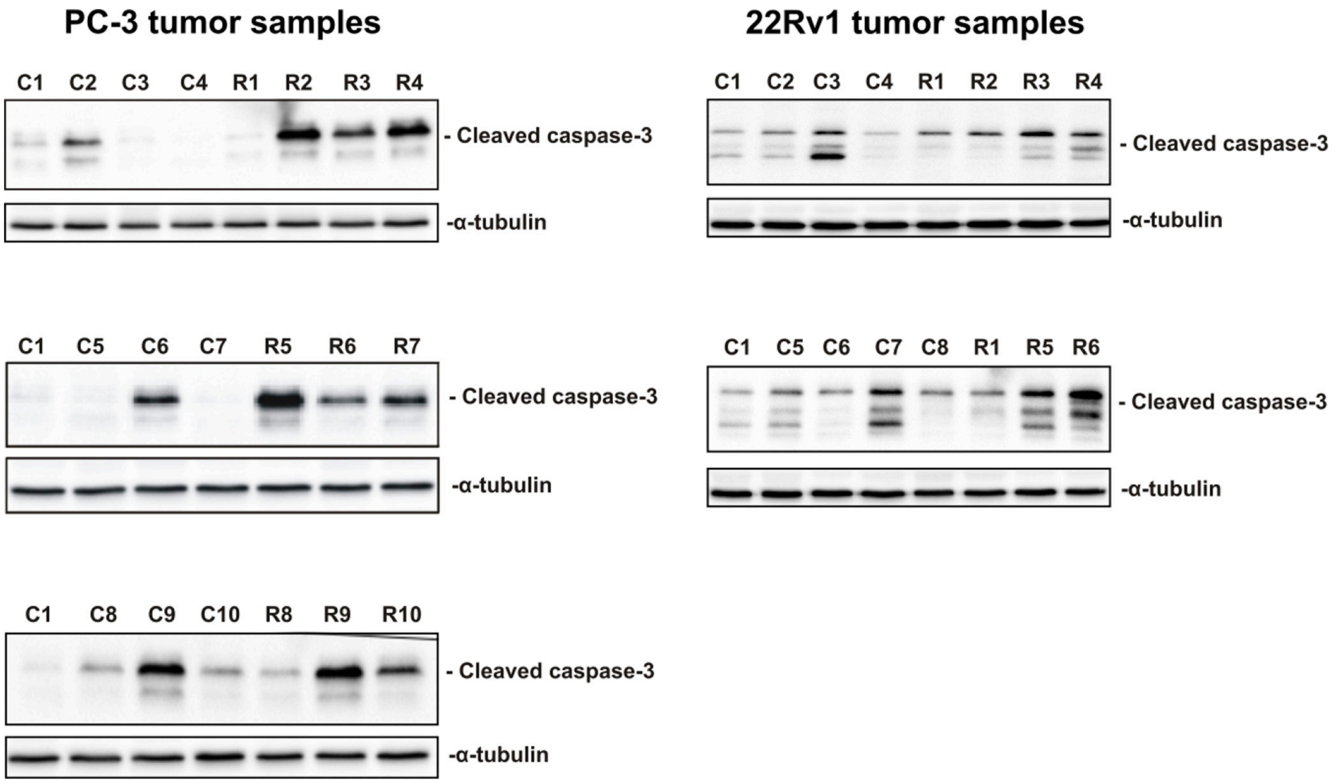

Supplementary Figure S3: Western blotting analysis of cleaved caspase-3 in the tumors. The tumor samples were homogenized and analyzed as described in the Materials and methods. The quantification of the signal was performed using the Image J Software (NIH, Bethesda, USA). The level of cleaved caspase-3 was normalized against loading control. The signals within each membrane were normalized against the same control sample (which was loaded on each gel) and therefore were compared within the different membranes and then compared. “C” – the sample from the control group, “R” – the sample from the Rhiz-treated group.
